# Assessing intake of spices by pattern of spice use, frequency of consumption and portion size of spices consumed from routinely prepared dishes in southern India

**DOI:** 10.1186/1475-2891-14-7

**Published:** 2015-01-11

**Authors:** Vasanthi Siruguri, Ramesh V Bhat

**Affiliations:** Food & Drug Toxicology Research Centre, National Institute of Nutrition ICMR, Hyderabad, 500007 India; Centre for Science, Society and Culture, M 11, Kakateeyanagar, Habshiguda, Hyderabad, 500007 India

**Keywords:** Spices, Dietary intake, Portion size

## Abstract

**Background:**

Measurement of dietary intake of spices is gaining significance because of recognition of their health promoting benefits as well as its use for risk assessment of contaminant exposures. Estimating intake of spices at the individual level, presents several challenges since various spices are used as an integrated part of a prepared food and consumed in amounts much smaller than other dietary components. The objective of the present study is to assess intake of spices at the household and individual level on the basis of pattern of spice use and portion size of spice consumed from routinely prepared dishes in Hyderabad city in Southern India.

**Methods:**

The study was conducted in 100 households in urban areas of Hyderabad city in India with the help of a spice intake questionnaire that was prepared to collect information on the pattern of spice use, frequency, and quantity of spice consumption of 17 spices routinely used in Indian cuisine. The quantity of spice intake was assessed by measuring portion size of spice consumed from the quantity of i) spices added in routinely prepared dishes and ii) the prepared dish consumed by an individual.

**Results:**

Based on the type of dish prepared and frequency of preparing the dishes, 11 out of 17 spices were found to be consumed by more than 50% of the households. Maximum number of spices was consumed at weekly frequencies. Red chillies and turmeric were the most frequently consumed spices by 100% of the households. The mean total intake of spices was observed to be higher through dishes consumed daily (10.4 g/portion) than from those consumed at weekly or monthly frequencies. Highest portion size intake was observed for chillies (mean 3.0 g; range 0.05-20.2 g) and lowest for nutmeg (mean 0.14 g; range 0.02-0.64 g) and mace (mean 0.21 g; range: 0.02-0.6 g).

**Conclusions:**

The study suggested that assessment of intake of spices varies with frequency of use of spices and type of dish consumed. Portion size estimations of spices consumed and the frequency of consumption of the spice containing dishes facilitates in quantifying spice intake at the individual level.

## Background

Spices play an important role as flavouring agents in the diet and are used throughout the world. Spices refer to the dried part of a plant that contain volatile oils or aromatic flavours such as, buds (cloves), bark (cinnamon), root (ginger), berries (black pepper), seeds (cumin, coriander) [1]. While consumption of spices is generally higher in Asian countries such as India, China, and Thailand, there has been an increasing trend in their intake in developed countries such as in Europe and the USA, because of changing food habits and preference for ethnic and spicy food [2, 3].

In recent times measurement of dietary intake of spices is gaining much significance as various phytochemicals present in spices have been recognized to have health promoting benefits and preventive role in chronic diseases [4, 5]. Measurement of spice intake as part of total diet studies or risk assessment for estimating contaminant intakes is also assuming considerable importance [6].

Methodology for measuring spice intake is just emerging and few studies have been conducted specifically to quantify spices intake at the individual level [5, 7–11]. Methods using 24-hour recalls, food records, or diaries, weighment of food items, and food frequency questionnaires (FFQ) have been used in these studies to quantify spice intake. Spices are consumed in amounts much smaller than other dietary components such as staple cereals, constituting 0.8-2.2% of the total dry matter content of the diet [4, 7]. Quantification of spice intake at the individual level presents several challenges as the frequency and quantity of intake varies with the type of spice, form in which it is used, quantity added to various dishes and the frequency of preparing and consuming such dishes. Estimation of portion size of spice consumed per eating occasion or frequency is emerging as a useful approach for quantifying spice intake [10, 11]. In the present study an attempt was made to assess dietary spice intake at the household and individual level by estimating the frequency and quantity of spice intake from routinely prepared dishes in southern India based on pattern of spice use and portion size of spice consumed. The results are discussed in comparison to the available data on spice intake in India and other countries.

## Methods

### Selection of spices and standardization of household measures

A total of 17 spices that are commonly used in Indian cuisine, were selected to assess their dietary intake. The scientific and common/local names of the selected spices are shown in Table 1. Since most spices are used in small quantities, the weights of each of the selected spices, whole and powdered, were initially standardized by using standard HH measuring spoons that measure 1 teaspoon, and 1/4^th^ teaspoon. A portable balance (Yamasa, Japan) with a measuring range of 1-100 g, was used for weighing small quantities of spices at the HH level, and was also standardized by measuring weights of spices that were weighed using HH measures. The weights obtained using HH measures and portable balance were further verified with an electronic balance (Denver Instruments Company), that had a weighing range of 0.1 to 250 g and sensitivity of 0.001 g.

**Table 1:** **Scientific and local names of selected spices evaluated in the study**

S. no	Name of spice	Scientific name	Local name
1	Red chillies	*Capsicum annuum* L. & *Capsicum frutescens* L.	*Lal mirch*
2	Turmeric	*Curcuma longa* L.	*Haldi*
3	Cumin seeds	*Cuminum cyminum* L.	*Zeera*
4	Coriander seeds	*Coriandrum sativum* L.	*Dhania*
5	Mustard seeds	*Brassica juncea* L.Czern	*Rai*
6	Fenugreek seeds	*Trigonella foenum-graecum* L.	*Mehthi*
7	Black pepper	*Piper nigrum* L.	*Kali mirch*
8	Cloves	*Syzygium aromaticum*	*Lavang*
9	Cardamom	*Elettaria cardamomum* Maton	*Ilaichi*
10	Cinnamon	*Cinnamomum zeylanicum* Breyn	*Dalchini*
11	Caraway seeds	*Carum carvi* L.	*Shahzeera*
12	Carom seeds	*Trachyspermum ammi* L.	*Ajwain*
13	Nutmeg	*Myristica fragrans*	*Jaiphal*
14	Mace	*Myristica fragrans*	*Japatri*
15	Fennel	*Foeniculum vulgare* Mill.	*Saunf*
16	Asafoetida	*Ferula asafoetida*	*Hing*
17	Star Anise	*Illicium verum*	*Anasphal*

### Spice intake survey

A spice intake questionnaire was prepared to collect the following information on the pattern of spice use and intake in each HH : i) type of spices used, ii) frequency of usage and intake with options of ‘daily’, ‘1’, ‘2’, or ‘3’ times per week, ‘1’ or ‘2’ times per month, ‘occasionally’, and ‘never’, iii) the quantity of spices used in routine dishes which included details of type of dishes prepared in which spices were added, the type and quantity of spices added per dish, and the frequency of preparing the dishes, and iv) the quantity of prepared dish consumed by an adult individual in order to calculate the portion size of the added spice consumed. The above questionnaire was pretested in 10 HHs and was found satisfactory for collecting the above information.

### Study population

The study population consisted of 100 urban HHs in selected locations in different metropolitan zones in Hyderabad city. The HHs belonged to different socio-economic groups, and to different religious groups such as Hindus and Muslims. The above pre-tested questionnaire was administered by a trained interviewer to each of the 100HHs to obtain information on frequency, portion size, and quantity of spice consumed at the individual level in each HH. The main individual performing cooking activities in the household was interviewed to elicit information on the pattern of spice use and intake as described in the questionnaire. For easy identification of various spices by the respondent, a chart showing samples of each of these spices was shown to the respondent while administering the questionnaire. The quantity of spices added to various dishes was measured using standardized measuring spoons and portable balance that were calibrated as described earlier.

### Assessment of spice intake

Spice intake was assessed for each of the spices consumed based on i) frequency of consumption of spices; ii) type and quantity of spices added to dishes routinely prepared and frequency of preparing the dishes, and iii) the portion size of the spice consumed based on the quantity of dish/dishes consumed by an individual (adult member) in the HH.

Frequency of consumption of spices was assessed based on the usage of spices by the HHs (expressed as number of HHs) with daily, weekly, and monthly options as described in the questionnaire. The type of spices added to various dishes was assessed by identifying regularly prepared dishes that included various spices in the HHs. The most frequently used spices in these dishes were identified by the number of HHs using them. The quantity of spice intake by an individual was assessed based on portion size of each spice consumed from the quantity of prepared dish/dishes consumed and expressed as i) spice intake per portion size of spice consumed from all dishes; ii) spice intake per portion size of each individual dish consumed; iii) spice intake per portion size of spice consumed at different frequencies of preparing the dishes (daily, weekly, or monthly). The quantities of spice intake were expressed as Mean ± Standard Deviation (S.D.), Median, 90^th^ and 97.5^th^ percentile values, and ranges (minimum and maximum).

## Results

### Type and frequency of spices used and consumed

Spice intake survey in 100 HHs showed that more than 50% consumed spices listed from 1–11 in Table 1, with 99 and 100% of the HHs consuming red chillies, and turmeric daily respectively (Table 2). Among the remaining spices that were consumed by less than 50% of the households, about 35-41% of the households consumed carom and fenugreek seeds, 31 and 25% of the households consumed nutmeg and mace respectively, and 24% consumed asafoetida. The least consumed spice was star anise (3% of the HHs).

**Table 2:** **Frequency of spice consumption by the HHs (Number of HHs)**

S. no	Spice	No. of HHs consuming	Frequency of consumption						
			Daily	Weekly			Monthly		Occas.
				1	2	3	1	2	
1.	R.chillies	100	99	60	45	35	0	8	0
2	Turmeric	100	100	54	41	40	6	17	0
3	Cumin	99	44	45	34	33	2	8	0
4	Cardamom	95	6	71	20	8	10	17	12
5	Cloves	90	1	65	16	6	5	9	1
6	Cinnamon	86	2	61	18	6	5	8	0
7	Coriander	85	30	38	20	9	3	4	0
8	Caraway	83	2	56	14	7	6	10	0
9	B. pepper	72	27	38	13	9	2	10	2
10	Fennel	69	40	6	5	0	0	2	16
11	Mustard	64	23	37	21	19	0	5	0
12	Fenugreek	41	7	21	8	2	2	3	1
13	Carom	35	3	11	3	1	5	3	10
14	Nutmeg	31	0	14	6	1	6	2	3
15	Mace	25	0	13	5	1	0	5	2
16	Asafoetida	24	12	9	3	3	0	1	1
17	Star Anise	3	0	0	2	0	0	1	0

Frequency of spice consumption: ‘1’, ‘2’, ‘3’ times/week, ‘1’, ‘2’ times/month.

Occas: Occasionally. R.chillies: Red chillies; B.pepper: black pepper; Cumin, coriander, Mustard, Caraway, Carom, and fennel refer to seeds.

### Type of dishes routinely prepared in the HHs

A total of 4 main dishes routinely prepared in the HHs were identified in which various spices were used which included, *Curry* (vegetable or non-vegetarian preparation), *Dhal* (legume preparation), *Chutney* (relish made by grinding vegetables, fruits, and assorted spices mostly chillies), and Rice preparations (*biryani*, fried rice, *kitchidi* (mix of rice, legumes, and assorted spices) (Table 3). Snacks, salad, and sweets were the other preparations that involved a variety of spices including red chillies, black pepper, cardamom, nutmeg, and mace. Table 3 shows the most frequently used spices in these dishes and are arranged in a descending order based on the number of HHs using them. Maximum number of spices were used in *Curry* and *Rice* preparations. The type of spices used varied with the type of preparations.

**Table 3:** **Type of spices used by the HHs in routinely prepared dishes**

Name of the dish							
***Curry***		***Rice***		***Chutney***		***Dhal***	
Spices used	No. of HHs	Spices used	No. of HHs	Spices used	No. of HHs	Spices used	No. of HHs
R.chillies	99	Cardamom	86	Cumin	88	Cumin	87
Turmeric	99	Cinnamon	77	Chillies	77	Chillies	85
Coriander	60	Cloves	70	Turmeric	64	Turmeric	83
Cardamom	43	Caraway	70	Mustard	49	Mustard	43
Caraway	42	Turmeric	64	Coriander	20	Asafoetida	16
Cloves	41	R.chillies	56	Asafoetida	5		
Cinnamon	40	B.pepper	40	Fenugreek	4		
B. pepper	25	Nutmeg	24				
Fenugreek	16	Mustard	20				
Asafoetida	9	Mace	14				
Cumin	7	Cumin	9				
Mustard	6	Fenugreek	4				
Mace	6	Carom	3				
Nutmeg	4	Asafoetida	2				
Carom	2	Star Anise	1				
Star Anise	2						

### Quantity of spice intake based on portion size of spice consumed

#### Spice intake based on portion size of spice consumed from all dishes

The mean, median, 90^th^ and 97.5^th^ percentile levels and ranges of the quantity of each spice intake calculated based on portion size of spice consumed from various dishes are shown in Table 4. A total number of 1905 portion sizes of spices consumed from all the dishes by adult member in 100 HHs were evaluated. Maximum number of portion sizes representing more than 10% of the total portion sizes belonged to chillies, turmeric, and cumin. The mean intakes were observed to be above 1 g/portion size for red chillies, cumin, coriander, caraway, and mustard seeds, and less than 1 g for other spices consumed. The highest mean and maximum quantity of intake per portion was observed for chillies (3 g and 20 g/portion respectively). Nutmeg and mace had the lowest mean intakes (0.14 g and 0.21 g/portion respectively). While the median intakes of majority of the spices were below 1 g, the 90^th^ and 97.5^th^ percentile values were above 1 g for 11 and 12 of the 16 spices respectively that were evaluated. The highest 90^th^ and 97.5^th^ percentile values were observed for chillies (6 and 11.1 g/portion respectively) and the lowest for nutmeg (0.2 and 0.32 g/portion respectively).

**Table 4:** **Quantity of spice intake per portion size of spice consumed from all dishes (g)**

Spice	No. of portion sizes	Mean	Median	90 ^th^ percentile	97.5 ^th^ percentile	Range
R.chillies	364 (19.1%)	3.0 ± 3.05	2.1	6	11.1	0.05-20.2
Turmeric	351 (18.4%)	0.71 ± 0.51	0.6	1.35	2.0	0.09-3.75
Cumin	198 (10.4%)	1.64 ± 1.59	1	3.2	6.0	0.04-10
Cardamom	170 (8.9%)	0.39 ± 0.54	0.25	0.6	1.4	0.01-4.24
Cloves	122 (6.4%)	0.34 ± 0.62	0.11	0.75	2.4	0.01-4.5
Mustard	122 (6.4%)	1.07 ± 0.9	0.75	2.5	3.5	0.15-6.0
Cinnamon	119 (6.2%)	0.77 ± 0.82	0.5	1.5	2.4	0.02-6.4
Caraway	110 (5.8%)	1.08 ± 1.01	0.7	2.3	3.7	0.08-5.52
B. pepper	108 (5.7%)	0.97 ± 1.05	0.5	3	3.6	0.04-4.4
Coriander	96 (5%)	1.37 ± 1.6	0.84	3.06	5.6	0.06-9.2
Asafoetida	41 (2.2%)	0.75 ± 0.76	0.4	1.5	1.6	0.12-4.5
Fenugreek	31 (1.6%)	0.82 ± 0.96	0.4	1.62	3.24	0.04-4
Nutmeg	30 (1.6%)	0.14 ± 0.11	0.13	0.2	0.32	0.02-0.64
Mace	23 (1.2%)	0.21 ± 0.13	0.2	0.4	0.4	0.02-0.6
Carom	20 (1%)	0.69 ± 0.58	0.3	1.5	1.5	0.19-2.4
Total	1905					

Portion size of each spice consumed: aggregate of intakes from curry, rice, chutney, dhal, and other dishes.

The distribution of the levels of spice intakes based on portion size showed that with the exception of chillies and cumin, more than 50% of the portion sizes consumed were below 1 g for the majority of spices (Table 5). Majority of portion size intakes of chillies (70%) were between 1-5 g and about 15% were above 5 g. More than 90% and 100% of portion size intakes of cloves and cardamom and of nutmeg and mace were below 1 g respectively.

**Table 5:** **Distribution of levels of spice intake based on portion size consumed**

Spice	Number of portion sizes	Level of intake (% of portion sizes)		
		<1.0	1.0-5.0	>5.0
R. chillies	364	15.1	69.5	15.4
Turmeric	351	78.3	21.7	-
Cumin	198	39.4	54.5	6.1
Coriander	97	57.7	35	7.2
Mustard	121	61.1	38.8	-
Cloves	122	92.6	7.4	-
Cardamom	170	93	7	-
Cinnamon	118	75.4	24.6	-
Caraway	109	63.3	36.7	-
B. pepper	108	67.6	32.4	-
Nutmeg	30	100	-	-
Mace	23	100	-	-
Fenugreek	31	71	29	-
Asafoetida	41	78	22	-
Carom	25	76	24	-

‘-‘ nil.

#### Spice intake based on portion size of spice consumed from individual dishes

When spice intake from individual dishes was evaluated it was observed that the number of portion sizes consumed was highest from *Rice* and *Curry* followed by *Chutney* and *Dhal* preparations (Table 6). The mean intakes were highest for chillies especially from *Chutney* and *Dhal*. Median intakes were observed to be above 1 g only for chillies, cumin, and coriander seeds. Highest 90^th^ percentile intake level was observed for chillies from *Dhal* (9 g) and lowest for nutmeg and mace (0.2 g) from *Rice*. The mean total spice intake was observed to be highest from *Curry* (10.4 g/portion) followed by *Chutney* (9.6 g/portion), *Rice* (8.8 g/portion), and *Dhal* (8.3 g/portion).

**Table 6:** **Quantity of spice intake per portion size of spice consumed from individual dishes (g)**

***Curry***					
Spice	No. of portion sizes	Mean ± S.D.	Median	90 ^th^ percentile	Range
Chillies	120	2.06 ± 1.24	1.8	3.6	0.06-7.2
Turmeric	120	0.67 ± 0.5	0.54	1.2	0.09-3.75
Coriander	58	1.43 ± 1.77	0.85	3.4	0.12-9.2
Cardamom	23	0.44 ± 0.38	0.3	0.84	0.08-1.42
Cinnamon	22	0.75 ± 0.74	0.5	1.44	0.02-3.4
Caraway	20	1.42 ± 1.14	0.92	2.8	0.3-4.32
Cloves	18	0.44 ± 0.44	0.2	0.9	0.04-1.5
B. pepper	18	1.27 ± 1.3	0.31	3	0.14-3.6
Fenugreek	17	0.87 ± 1.05	0.4	1.2	0.16-4
Asafoetida	13	0.5 ± 0.38	0.4	0.9	0.2-1.5
Cumin	7	0.55 ± 0.28			0.04-1.5
Total		10.4 ± 9.22			
		***Rice***			
**Spice**	**N**	**Mean ± S.D.**	**Median**	**90** ^**th**^ **percentile**	**Range**
Chillies	56	2.38 ± 2.17	1.8	5	0.05-12.4
Turmeric	64	0.71 ± 0.49	0.6	1.35	0.1-3
Cloves	65	0.39 ± 0.8	0.1	1.2	0.03-4.5
Cardamom	70	0.47 ± 0.75	0.26	0.6	0.04-4.24
Cinnamon	62	0.94 ± 0.99	0.6	1.8	0.02-6.4
Caraway	59	1.14 ± 1.09	0.9	2.3	0.11-5.52
B.pepper	30	1.11 ± 1.18	0.75	3	0.1-4.4
Mustard	20	1.27 ± 1.27	0.83	2.25	0.4-6
Nutmeg	17	0.15 ± 0.14	0.13	0.17	0.04-0.64
Mace	12	0.19 ± 0.09	0.2	0.23	0.07-0.4
Total		8.75 ± 8.97			
***Chutney***					
**Spice**	**N**	**Mean ± S.D.**	**Median**	**90** ^**th**^ **percentile**	**Range**
Cumin	88	1.94 ± 1.89	1.2	4.1	0.13-10
Chillies	77	4.15 ± 4.15	2.4	8.8	0.5-20.2
Turmeric	64	0.74 ± 0.48	0.6	1.5	0.12-2.25
Mustard	49	0.99 ± 0.77	0.75	2	0.15-3.6
Coriander	18	1.76 ± 1.29	1.4	3.06	0.32-4.6
Total		9.58 ± 8.58			
***Dhal***					
**Spice**	**N**	**Mean ± S.D.**	**Median**	**90** ^**th**^ **percentile**	**Range**
Chillies	85	4.18 ± 3.75	2.88	9	0.36-18.72
Turmeric	83	0.79 ± 0.57	0.6	1.5	0.12-3.2
Cumin	87	1.59 ± 1.35	1.2	2.6	0.12-7.5
Asafoetida	22	0.6 ± 0.4	0.4	1.2	0.12-1.62
Mustard	43	1.16 ± 0.92	0.9	2.7	0.15-3.6
Total		8.32 ± 6.99			

#### Spice intake based on portion size of spice consumed at different frequencies

Table 7 shows the quantity of spice intake per portion size of spice consumed from various dishes prepared at different frequencies. Maximum intakes of spices were observed at weekly frequencies. Intake of cloves, cardamom, cinnamon, caraway seeds, nutmeg, and mace was observed to be more at weekly intervals and were below 1 g. The mean intakes above 1 g were observed for chillies, and cumin, at all three frequencies and for coriander and mustard seeds at weekly and monthly frequencies.

**Table 7:** **Quantity of spice intake per portion size consumed from dishes prepared at different frequencies (g)**

Spice	Frequency of dishes prepared					
	Daily		Weekly		Monthly	
	N	Intake mean ± S.D (Median)	N	Intake mean ± S.D (Median)	N	Intake mean ± S.D (Median)
Chillies	157	2.1 ± 1.3 (1.8)	186	3.8 ± 3.88 (2.32)	21	3.0 ± 1.92 (2.25)
Turmeric	156	0.6 ± 0.46 (0.66)	171	0.82 ± 0.53 (0.72)	24	0.73 ± 0.46 (0.6)
Cumin	61	1.22 ± 1.14 (1.0)	125	1.85 ± 1.78 (1.2)	12	1.66 ± 1.07 (1.4)
Coriander	27	0.8 ± 0.65 (0.5)	62	1.5 ± 1.74 (0.84)	7	2.47 ± 2.22 (3.5)
Mustard	34	0.55 ± 0.31 (0.5)	81	1.24 ± 0.95 (1.0)	7	1.65 ± 1.2 (1.12)
Cloves	-	-	105	0.33 ± 0.62 (0.11)	16	0.48 ± 0.68 (0.12)
Cardamom	6	0.67 ± 0.64 (0.63)	134	0.37 ± 0.57 (0.25)	18	0.5 ± 0.36 (0.32)
Cinnamon	-	-	102	0.74 ± 0.83 (0.5)	15	1.03 ± 0.83 (0.8)
Caraway	-	-	94	1.1 ± 1.07 (0.7)	18	1.22 ± 0.89 (0.92)
Black pepper	28	0.69 ± 0.87 (0.5)	64	1.07 ± 1.16 (0.5)	16	1.07 ± 0.81 (0.72)
Nutmeg	-	-	23	0.11 ± 0.07 (0.11)	7	0.22 ± 0.19 (0.14)
Mace	-	-	18	0.19 ± 0.13 (0.16)	5	0.27
Asafoetida	17	0.37 ± 0.16 (0.4)	16	0.72 ± 0.49 (0.5)		-
Fenugreek	-	-	25	0.68 ± 0.65 (0.4)	4	2.04
Carom seeds	-	-	13	0.69 ± 0.64 (0.45)	5	0.66

N: No. of portion sizes.

## Discussion

Measurement of spice intake at the individual level is a challenging and more difficult task than measuring intake of staples like cereals and other foods, which are consumed in relatively larger quantities and on daily basis. Food consumption survey methods such as food frequency questionnaires supplemented with dietary recall, weighed records, and portion size estimations have been used in various studies to assess dietary spice intake [12–14]. The present study made an attempt to assess dietary spice intake and quantify it at the individual level on the basis of pattern of spice use in the diet and portion size of spice consumed from routinely prepared dishes in selected urban HHs in a southern Indian city.

The study showed that among the 17 spices investigated, 11 spices were found to be frequently used and consumed by majority of the HHs surveyed. Spices were consumed both as powdered and whole spice either individually or as a mixture of 2 or more spices, depending on the dish prepared. The use and consumption of spices is known to be high in Indian cuisine. A review of number of spices used in 36 countries through traditional cook books revealed a mean number of 9.3 and 6.5 spices in meat- and vegetable –based recipes in India while in European countries it ranged from 1.6-4.5 spices in meat based and 0.6-4.2 spices in vegetable based recipes respectively [15]. In Norway, Carlsen *et al.* found that out of 27 different herbs and spices investigated only 8 were consumed by 1/3 or more of the population [11]. In the present study, a minimum of 5 and a maximum of 11 spices were used in the routine dishes evaluated with chillies and turmeric being the most frequently used spices. Earlier studies in India showed that frequency of consumption of red chillies and turmeric is higher than most other spices which is consistent with the observations in the present study [7, 8, 5, 9] (Table 2). Ferucci et al. observed regional differences in per capita spice consumption with the southern regions consuming more number of spices as compared to northern and western regions in India [5]. In European countries such as Norway, intakes of spices ranged from 0.8 to 14.7 times per month which is relatively lower than the high frequency of spice consumption observed in the present study [11].

The use and consumption of spices in the HHs also varied with the type of dish prepared which was also observed in studies from Thailand [10]. In most of the routine dishes consumed in North-eastern Thailand, use of chillies was common in all the dishes. In the present study it was observed, that chillies and turmeric were used in all the routine dishes particularly in *Curry*, *Chutney*, and *Dhal* preparations, whereas cloves, cinnamon, cardamom, caraway seeds, and black pepper were the dominant spices used in *Rice* dishes by majority of the HHs (Table 3). Other spices such as cumin, coriander, and mustard seeds, were added mostly in *Chutney* and *Dhal* preparations and their usage in other dishes was limited. Spices such as fennel seeds were consumed as an after meal digestive in majority of the HHs (85%) and their inclusion as a spice in various preparations was observed to be limited in the present study. The observations in the present study although limited to a selected region in southern India assume relevance in the context of understanding use of spices in various preparations that may impact quantity of intake of various spices based on the frequency of consuming a particular dish or preparation.

In the present study quantity of spice intake was assessed from the portion size of individual spices consumed from routine dishes prepared in the HHs. Few studies are available on portion size intakes of individual spices and have been reported from Thailand and Norway for spice and herbs [10, 11]. In Thailand, the portion eaten per meal of each relevant spice or herb from 4 most commonly consumed dishes in north eastern Thailand were determined by measuring the amount of spice added to each dish before preparation, the weight of the prepared dish, and individual portion eaten of each dish. In the present study, the portion size of spice consumed was determined similarly but the details on the quantity of spices added and prepared dish consumed was recorded by weighing the quantities recalled by the respondent who was involved in preparing the dishes than actual observation during cooking. The weights of spices added to various dishes and portion size consumed were also verified through a repeat survey in selected HHs by 24 hr recall (data not shown), and found to be comparable. In the Norway study, the number of eating occasions or frequencies has been used together with food databases to estimate intakes of portion size [11]. The mean and median portion sizes per eating occasion estimated for 5 individual spices and 3 spice blends ranged from 0.5 -1.3 g and 0.2-0.9 g respectively. In the present study the number of portion sizes evaluated was relatively higher than that reported in the above studies with mean intakes ranging from 0.14 to 3 g/portion (Table 4). Large variations exceeding 200% in the mean intakes were observed for each spice which could be attributed to variation in amount of individual spices used, the quantity of dish consumed, and also individual preference. Thus, intakes ranged from as low as 0.01 for cloves and cardamom to a high of 20 g for chillies. Majority of portion sizes of spice consumed were represented by chillies (19%) followed by turmeric (18.4%) and cumin (10.4%) thus indicating the high frequency of consumption of these spices. The mean intakes of chillies and turmeric based on portion size were comparable to the per capita intakes of these spices reported from India by other investigators which were in the range of 2.0-3.0 g and 0.2-0.87 g/day/person respectively [7, 9, 5]. These observations further support the high frequency and quantity of intake of chillies and turmeric. Higher intakes exceeding 5 g/day of chillies were observed in rural regions in India [8]. In the present study majority (70%) of the portion sizes of chillies consumed were in the range of 1-5 g and about 15% were above 5 g (Table 5). High intake of chillies has been reported from Thailand and Mexico (5 and 20 g/person/day respectively) [16]. In Thailand, portion size intakes of dried chilli pepper were observed to range from 0.7-2.2 g from various dishes particularly from chilli sauce or paste [10]. In the present study chilli intakes per portion size were found to be highest from *Chutney* and *Dhal* preparations (4 g). Pradeep et al. reported spice intake data based on Consumption Units per day for 11 spices of which intakes of chillies, turmeric, and coriander seeds were comparable with the portion sizes obtained for these spices in the present study [9].

Data on intake of other spices such as cloves, cardamom, cinnamon, nutmeg, and mace is less documented as compared to that of chillies. In the present study, portion sizes of cloves, cardamom, cinnamon, nutmeg, and mace represented less than 10% of the total portions indicating the lower frequency of consumption of these spices (Table 4). Majority of these spices had intake levels below 1 g indicating the use of low quantity of these spices in various dishes as compared to that of chillies (Table 5). Ferucci et al. reported per capita monthly median intake levels of 3.1 g for cloves and 0.17- 4.6 g for cardamom in different regions of India. These levels are higher than the mean portion size intake of cloves and cardamom at monthly frequency observed in the present study (0.5 g) [5]. These differences may be attributed to the different methods of quantifying the spice intakes as measurement of portion size is based on the actual quantity of spice consumed from a dish in an eating occasion by an individual while per capita intake may represent average consumption at the household level [17]. In Norway, the mean portion size intake of cinnamon was 1.3 g per eating occasion which is higher than that observed for cinnamon in the present study (0.77 g, Table 4) [11]. High intake of cinnamon has been documented in Europe with maximum intakes up to 0.22 g/kg body weight through consumption of rice pudding in children [18]. Cinnamon is used in a variety of bakery and confectionery items in European countries whereas in India it is used along with other spices such as cloves and cardamom for *Rice* or *Curry* preparations as shown in the present study. Intake of black pepper in the present study was comparable to the level reported in Norway when assessed from individual dishes such as *Curry* (1.27 vs 1.3 g/portion). Black pepper is used considerably in Indian cuisine particularly as part of various spice blends and also in salads. In the present study majority (40%) of the total portion sizes of black pepper were from intake of salad (data not shown). The mean portion size intake of black pepper (0.97 g, Table 4) was observed to be higher than the per capita level of intake reported by other studies [8, 9]. The mean intake levels for nutmeg and mace were found to be the lowest among the spices assessed in the present study indicating the occasional and low quantity of use of these spices in the dishes evaluated. The per capita intake of nutmeg and mace consumption estimated based on food production statistics is reported to be in the range of 0.1 g in European countries [19] which is comparable to the portion size estimate observed in the present study (0.14-0.23 g/portion) (Table 4).

It was observed in the present study that the quantity of spice intake based on portion size varied with the frequency of consuming a particular dish that contained the spice. The portion size intakes of spices were generally higher at weekly frequencies as more number of spices was used in dishes prepared at weekly frequencies than in those prepared daily. For example chilli and turmeric had maximum number of portion sizes from *Curry* that is prepared daily in almost all the HHs. However, mean intake of chilli was highest from *Chutney* and *Dhal* (4.2 g/portion) and a maximum portion size intake of 20 g was also observed from these dishes which were prepared mostly at weekly frequencies. The high 90^th^ and 97.5^th^ percentile values for chillies (6.0 and 11.1 g/portion respectively) were also observed from chutney and dhal preparations (Table 4 and 6). Similarly intake of cloves, cardamom, cinnamon, caraway seeds, nutmeg and mace was observed to be mostly from *Rice* dishes which were prepared at weekly frequencies. These observations assume relevance for quantifying spice intake since all spices are not consumed on a daily basis and intake of individual spices varied with frequency of consumption of dishes. These observations have been studied to a limited extent earlier. Thus, in the present study the mean total intake of spices was observed to be highest through *Curry* as it was consumed daily (10.4 g/portion) followed by *Chutney* (9.6), *Rice* (8.8), and *Dhal* (8.3) which were consumed mostly at weekly frequencies (Table 6). These levels are lower than the levels reported earlier from India where the mean total spice intake per person per day ranged from 10 g to 29 g [7–9]. In these studies intake of all spices was estimated on a daily basis without considering frequency of consumption of individual spices. In Thailand portion size intake of total spices and herbs on an average from habitual dishes was estimated to range from 4.9 g −26.1 g. In Norway, the median estimates of total herb and spice consumption was found to be 2.7 g/person/day. In the present study, portion size intakes of only spices were estimated and herbs were not investigated and were observed to be higher than the total intakes reported in these studies. This may be attributed to higher number of spices used per dish evaluated in the present study.

## Conclusions

The approach for assessment of dietary intake of spices need to be different than that used for staples since spices are used for flavouring foods in small quantities as integral part of diets. The intake of individual spices varies considerably between different countries, geographic regions within the same country, and also with different dietary cuisines within the same region. The present study made an attempt to explore measurement of portion size of spice consumed as a useful approach for quantifying spice intake at the individual level. Portion size estimations together with data on frequency of intake of individual spices provide a reasonably good quantitative estimate of spice intake than with frequency of intake alone. Identifying the type of dishes that use various spices and assessing the frequency of preparing and consuming such dishes facilitates quantification of spice intake at the individual level. The results of the present study indicated that few spices such as chillies are consumed in higher amounts than others like nutmeg, which were attributed to difference in type of dishes and frequency of preparing and consuming them. Although the study was limited to a small number of HHs in one city within India, it seems to be a useful approach for estimating spice intake, since it is based on pattern of spice use and portion size estimations. The approach would find considerable utility not only for estimating intake of bioactive principles, or micronutrients but also for risk assessment of chemicals or food contaminants such as aflatoxins that may be consumed through spices.
